# Asymmetry in host and parasitoid diffuse coevolution: when the red queen has to keep a finger in more than one pie

**DOI:** 10.1186/1742-9994-2-4

**Published:** 2005-03-01

**Authors:** Laurent Lapchin, Thomas Guillemaud

**Affiliations:** 1 "Biologie des Populations en Interaction", UMR 1112 "Réponse des Organismes aux Stress Environnementaux", Inra/Unsa, 400 route des Chappes, BP167 06903 Sophia-Antipolis cedex, France

## Abstract

**Background:**

Coevolution between pairs of antagonistic species is generally considered an endless "arms race" between attack and defense traits to counteract the adaptive responses of the other species.

**Presentation of the hypothesis:**

When more than two species are involved, diffuse coevolution of hosts and parasitoids could be asymmetric because consumers can choose their prey whereas preys do not choose their predator. This asymmetry may lead to differences in the rate of evolution of the antagonistic species in response to selection. The more long-standing the coevolution of a given pair of antagonistic populations, the higher should be the fitness advantage for the consumer. Therefore, the main prediction of the hypothesis is that the consumer trophic level is more likely to win the coevolution race.

**Testing the hypothesis:**

We propose testing the asymmetry hypothesis by focusing on the tritrophic system plant/aphid/aphid parasitoid. The analysis of the genetic variability in the virulence of several parasitoid populations and in the defenses of several aphid species or several clones of the same aphid species could be compared. Moreover, the analysis of the neutral population genetic structure of the parasitoid as a function of the aphid host, the plant host and geographic isolation may complement the detection of differences between host and parasitoid trophic specialization.

**Implications of the hypothesis:**

Genetic structures induced by the arms race between antagonistic species may be disturbed by asymmetry in coevolution, producing neither rare genotype advantages nor coevolutionary hotspots. Thus this hypothesis profoundly changes our understanding of coevolution and may have important implications in terms of pest management.

## Background

Coevolution is the result of reciprocal selective pressures exerted by interacting species. Many studies have been devoted to the hypothesis of an endless "arms race" between antagonistic species, in which each species develops escalating attack and defense traits to counteract the adaptive responses of the other species. In reference to Lewis Carroll's book "Through the Looking Glass", Van Valen [1] named this model of coevolution "the Red Queen Hypothesis" (RQH) because, even in a constant physical environment, interacting species must evolve continuously to maintain their position. The RQH can be seen as an arms race between "resistance" and "virulence" where, following recent reviews on coevolution (e.g. [2]), "resistance" is the target's ability to survive attacks by the consumer, and "virulence" is the consumer's ability to defeat the target's defenses.

The RQH was initially developed in the context of multiple species interactions, to account for the constant probability of species extinction. However, as modeling the coevolution of many species involves a number of difficulties, later studies based on the RQH have mostly been limited to interactions between pairs of species. From this restricted situation, two main predictions can be made: first, arms races induce an advantage of rare genotypes of which resistance or virulence is more efficient and thus, frequency-dependent fluctuations of resistance and virulence may be predicted. Second, a geographic view of the coevolutionary process suggests a dynamic mosaic structure, with local and temporary "hot spots" of antagonistic species coevolution [3].

However, for plant-pathogen interactions [4] and for animal host-parasite interactions [5-7], empirical observations and experimental tests of RQH have not given entirely convincing results. For instance, there is considerable evidence of genetic variation for resistance, but parasite-driven genetic change in resistance has never been observed directly [8]. Conversely, in certain systems of interacting species, matching genetic diversity, which is expected under the RQH, is lacking [9]. Another study [2] has suggested that, for a given host-parasitoid association, the rank order of survival of different host strains exposed to different parasitoid strains remains constant. This lack of frequency dependence has been interpreted as evidence against the RQH.

Nevertheless, the RQH continues to lie at the heart of the debate concerning the coevolution of antagonistic species, probably because of the lack of plausible alternative hypotheses. Although metapopulation structures or time lags between the responses of antagonistic species to selective pressures have been evoked as reasons for the lack of clear experimental evidence in favor of the RQH [10], the additional hypothesis that is considered the most satisfactory relates to the cost of resistance [11]. Defense is thought to carry costs when it is not needed, i.e. the fitness of resistant individuals in the absence of enemies is reduced compared to susceptible individuals. Following this hypothesis, the arms race is constrained by the resistance cost and thus resistance and virulence of antagonistic species reach fixed levels that can be considered optimal. This hypothesis may result in a low level of genetic polymorphism of resistance and virulence in spatially restricted areas. In contrast, resistance and virulence are expected to be polymorphic on a larger spatial scale if, because of ecological factors, the densities of antagonistic species are spatially heterogeneous. However, although resistance costs have often been demonstrated, expected geographical patterns of resistance and virulence are not observed: antagonistic species densities are not clearly spatially associated with resistance and virulence variability levels [2]. Thus, although taking account the resistance cost hypothesis is a useful addition to Red Queen arms race models, this theoretical view of coevolutionary process does not provide predictions that fit well with most situations observed in the field [10,11].

## Presentation of the hypothesis

Adaptive responses to selective pressures exerted by antagonistic organisms are not necessarily the same for the target species and the consumer species. There is a first level of potential evolutionary asymmetry between them because the failure of virulence for a consumer is a delay in fitness acquisition whereas the failure of defense for a prey is the loss of its entire fitness. Such a difference was underlined by Dawkins [12] noting Aesop's fable of the hare outrunning the fox, because the former runs for life whereas the latter runs for a meal. The opposite asymmetry has been suggested in the case of host-parasitoid relationships [2,13]: all consumers are obliged to overcome the defenses of their target whereas not all targets suffer attacks from consumers.

Extending the field of interest to interactions and coevolution between more than two species (i.e. diffuse coevolution) may offer new perspectives. Considering the reciprocal selective pressures exerted by many species leads us to take into account the specificity of virulence and resistance. Until now, few works have been devoted to this subject (but see [14]). However, when considering diffuse coevolution, there is a second level of potential asymmetry because a consumer may choose its targets, whereas targets cannot be certain which of its enemies will attack it.

A recent model described the evolution of resistance of one host subjected to the attacks of two types of parasitoids differing in their virulence and specificity (different genotypes of a species or different species) and the evolution of virulence of one parasitoid attacking two types of hosts differing in their resistance and specificity [15]. The results suggested that the level of specialization of resistance traits was not affected by the total probability of being attacked. They also suggested that, if the probabilities of encounters fluctuate and differ between trophic levels, generalist traits of resistance and partially specialist traits of virulence are favored. Finally, this model showed that fluctuating host encounter probabilities across or within generations will promote a partial specialization of the parasitoid virulence rather than a total one.

The asymmetry hypothesis (AH) may lead to differences in the rate of evolution of the antagonistic species in response to selection. For a specialist consumer capable of target choice, chosen targets constitute a more or less "constant environment". On the other hand, for a generalist defender, the diversified, facultative and fluctuating attacks by a set of enemies constitute a "variable environment". The constancy of the environment may lead to the faster adaptation of specialists (mostly consumers under AH) than of generalists (mostly targets under AH) [16]. Thus, under the asymmetry hypothesis, the evolution of host defense traits is likely to be slower than the evolution of parasitoid virulence traits. The more long-standing the coevolution of a given pair of antagonistic populations, the higher should be the fitness advantage for the consumer. Therefore, the main prediction of AH is that the consumer trophic level is more likely to win the coevolution race [17]. This may account for the paradox described by Holt & Hochberg [18] – the lack of clear published examples of an increase in host resistance after biological control using parasitoids, despite the potentially strong selective pressure associated with parasitism.

The asymmetry hypothesis seems to fit well with numerous data concerning geographic structures published in the literature (reviewed in [15] but see also [19]). In most of these studies, the interaction trait under consideration is the capability of *Drosophila *species to encapsulate parasitoid eggs. In this case, it can be also noted that the resistance trait is not specific (artificial eggs are encapsulated as well as parasitoid ones) when virulence traits (reviewed in [20]) are diversified and specialized (i.e., escaping the host's immune response, just swamping the host with virus-like particles, or mimicking host tissue on the egg surface).

## Testing the hypothesis

We propose a dedicated test of the hypothesis of the specialization of the parasitoid virulence and the absence of specialization in the defense of the aphids, against the RQH. The study will focus on the tritrophic system plant/aphid/aphid parasitoid. Two complementary experimental approaches could be considered: A- The analysis of the genetic variability of virulence and defense of the parasitoid *Lysiphlebus testaceipes *and the aphid *Aphis gossypii*. To measure this variability, several populations of the parasitoid collected on different clones of the aphid will be confronted to different clones of this aphid. The different clones of the aphid will be confronted to the different parasitoid populations. This system that considers intra specific genetic variability for each trophic level will be used to evaluate the fitness of the consumers and of the targets in the different parasitoid /aphid combinations. An important point is that virulence will be estimated from the success rate of parasitism (i.e. the production of offspring) whereas defense will be estimated through the measure of the aphid fitness, i.e. the number of offspring produced by the aphid whatever the outcome of the parasitism (success or failure). Two key factors of the host fitness will be considered: 1) the host survival rate in case of parasitism failure (an aphid may die or not, even if the parasitism does not result in parasitoid offspring production); and 2) the number of offspring produced by the host after parasitism: after a parasitoid's sting, the host may produce offspring until mummification in case of parasitoid embryo development or until a variable date in the case of parasitism failure. B- The analysis of the neutral population genetic structure of the parasitoid as a function of the aphid host, the plant host and geographic isolation. The specialization of *L. testaceipes *on different aphid clones can lead to a neutral genetic differentiation of the parasitoid as a function of the host and the plant because the reproduction of the parasitoid occurs soon after the emergence of the adults from their host. The genetic differentiation between populations of the parasitoid sampled from different aphid clones and from different plant species could be evaluated and compared to a putative effect of isolation by distance.

This test may allow local verification or rejection of the predictions of the AH (specialization of the parasitoid and generalism of the host). When performed several times on animals from diverse geographic origins, this should eventually allow rejection of the classical interpretation of RQH and the host spots theory of coevolution [3].

## Implications of the hypothesis

The consequences of the asymmetry hypothesis are important. Genetic structures induced by the arms race between antagonistic species may be disturbed by asymmetry in coevolution, producing neither rare genotype advantages nor coevolutionary hotspots [17]. However some consequences of the AH are compatible with the resistance cost or the Red Queen hypotheses. Under the AH, a low variability in generalist resistance level, as expected under the hypothesis of regulation by the cost of resistance, would be selected for locally by the global pressure exerted by all the species of the upper trophic level. Also, higher levels of local variability in consumer specialist virulence would be selected, as in situations for which the classical RQH holds true.

The AH cannot be applied to every situation. In the case of host-parasitoid associations, the high level of genetic variability in resistance observed within some local host populations [20,21] is better explained by RQH if one dominant parasitoid species is present rather than a complex of species. Moreover, as generalism can be seen as a bet-hedging response to unpredictable environmental variations [15], the advantages of specificity or generalism depend on the level of such fluctuations. One can predict that host defenses will be less generalist when facing more stable enemy communities. Another key point deals with the supposed difference of speed in evolutionary responses between generalist and specialist traits. It may depend on other important biological factors like the discrepancy between population sizes, generation times and the reproductive mode (e.g. a sexual parasitoid versus an asexual aphid) or the genetic make up (e.g. an haplo-diploid parasitoid versus a diploid aphid) of the considered species. The relative speed of evolutionary responses of antagonistic species has thus to be evaluated in each biological model studied.

Asymmetry may be suspected in cases involving successive trophic levels other than host-parasitoid associations, such as plant-insect or parasitoid-hyperparasitoid combinations, as soon as individuals of the upper trophic level can choose their target. However, random attacks, such as those due to plant pathogens, may favor more generalist traits of virulence, but the specificity of consumers, and therefore asymmetry, may be restored through indirect choices: pathogens transmitted by vectors may use or manipulate the specificity traits of the vector. Large sets of species interactions may thus lead to asymmetric coevolution.

The AH may have consequences in terms of pest management. For instance, it is generally thought that variations in parasitism outcome result from a variability of host resistance due to the selection for higher resistance [2,22]. However, under the AH, virulence of the parasitoid is expected to evolve faster than does the resistance of the host. Therefore, the asymmetry hypothesis implies that variations in parasitism outcome more probably result from variability in parasitoid virulence. Asymmetry could also help to understand some surprising resistance-virulence patterns issuing from biological control. For instance, in the U.S.A., the pea aphid *Acyrthosiphon pisum *is parasitized by the hymenopteran *Aphidius ervi *and is specialized on different plant species. Hufbauer & Via [23] observed that pea aphids that are specialized on alfalfa are successfully parasitized less often than are pea aphids specialized on clover. Hufbauer [24] also observed that parasitoids collected from alfalfa and clover fields do not differ in their ability to overcome pea aphid resistance. They concluded that alfalfa host population is more resistant to *A. ervi *than is the clover host population. If the association they studied dealt with two aphid and one parasitoid populations, under the AH, virulence specialization rather than resistance level variations can explain the observations: the parasitoid population is specialized on the clover host population, but keeps a partial virulence on the alfalfa aphid biotype. This suggests that short-term parasitoid specialization may be a key factor in biological control efficiency. For instance, consumers introduced to control a pest could rapidly specialize against non-target hosts.

The asymmetry hypothesis thus provides food for thought concerning diffuse coevolution and could be applied to domains beyond host-parasitoid coevolution. Similar thoughts may be applicable to the durability and efficiency of plant resistance or immunological responses to diseases transmitted by vectors. Its theoretical implications and its consequences in terms of population management are potentially important and remain unexplored.

## List of abbreviations

RQH: Red Queen Hypothesis

AH: Asymmetry Hypothesis

## Authors' contributions

Both authors have been involved in the elaboration of the hypothesis and the evaluation of its consequences, and in the drafting of the paper.
